# Supplementary material for: Adapterama I: universal stubs and primers for 384 unique dual-indexed or 147,456 combinatorially-indexed Illumina libraries (iTru & iNext)
Source: PeerJ. 2019 Oct 11;7:e7755. doi: 10.7717/peerj.7755 (PMC6791352; doi:10.7717/peerj.7755)

Double Index 2-Step Library Method

Input DNA sample

1) Attach application-specific stubs

2) Use limited cycle PCR to add indexes and extend adapters

Full-length & double indexed

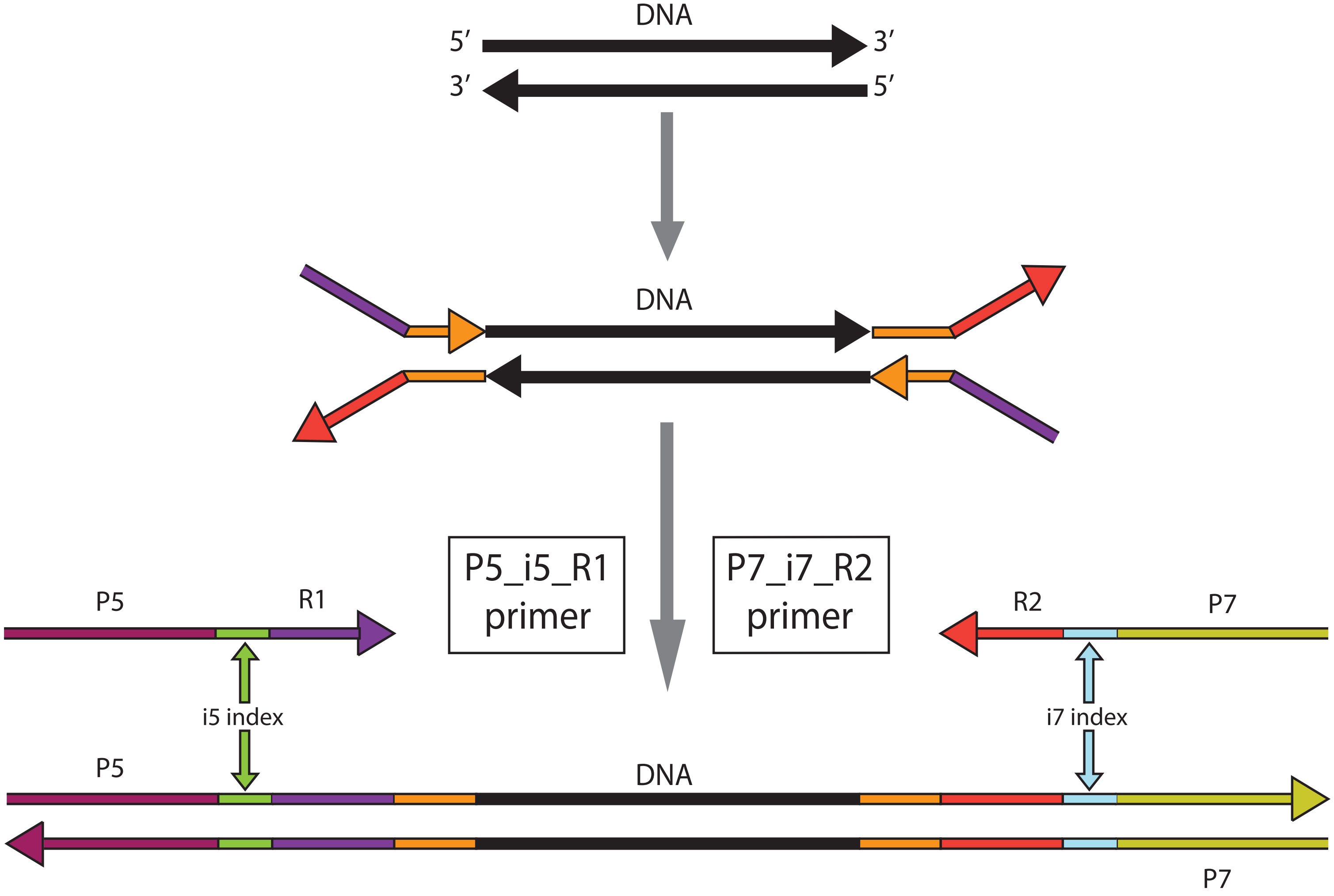

Supplement: Figure S1 — Adapterama is an integrated system of constructing libraries for next-generation DNA sequencing, whether by the iTru approach or iNext approach. The overall process for constructing dual-indexed Illumina libraries is depicted here. A variety of DNA inputs and application-specific processes are used to attach adapter stubs to the DNA of interest (black). In this illustration, we show a Y-yoke stub, but other configurations are possible and will be demonstrated in subsequent papers in the Adapterama series. Limited-cycle PCR is then used to extend the adapters, making them fully functional for sequencing on Illumina instruments and also adding dual indexes. The P5 (maroon) and P7 (light green) regions on the molecule are complementary to oligonucleotides present on Illumina flow cells, allowing for hybridization and clonal amplification. The i5 (dark green) and i7 (light blue) indexes can be used for multiplexing. The Read 1 (R1, violet) and Read 2 (R2, red) primer binding sites are complementary to the sequencing primers, enabling sequencing of the library molecules on the flow cell. The R1 and R2 primer binding sites also contain regions with identical sequence (shown in orange) that are used to facilitate the Y-yoke adapters. Thus, the full R1 and R2 sequences include the regions in orange (see Fig. 2). The color scheme matches Figure 1 and is used throughout, except as noted. [file peerj-07-7755-s001.pdf]
