# Supplementary material for: Adapterama I: universal stubs and primers for 384 unique dual-indexed or 147,456 combinatorially-indexed Illumina libraries (iTru & iNext)
Source: PeerJ. 2019 Oct 11;7:e7755. doi: 10.7717/peerj.7755 (PMC6791352; doi:10.7717/peerj.7755)

iNext Library Method

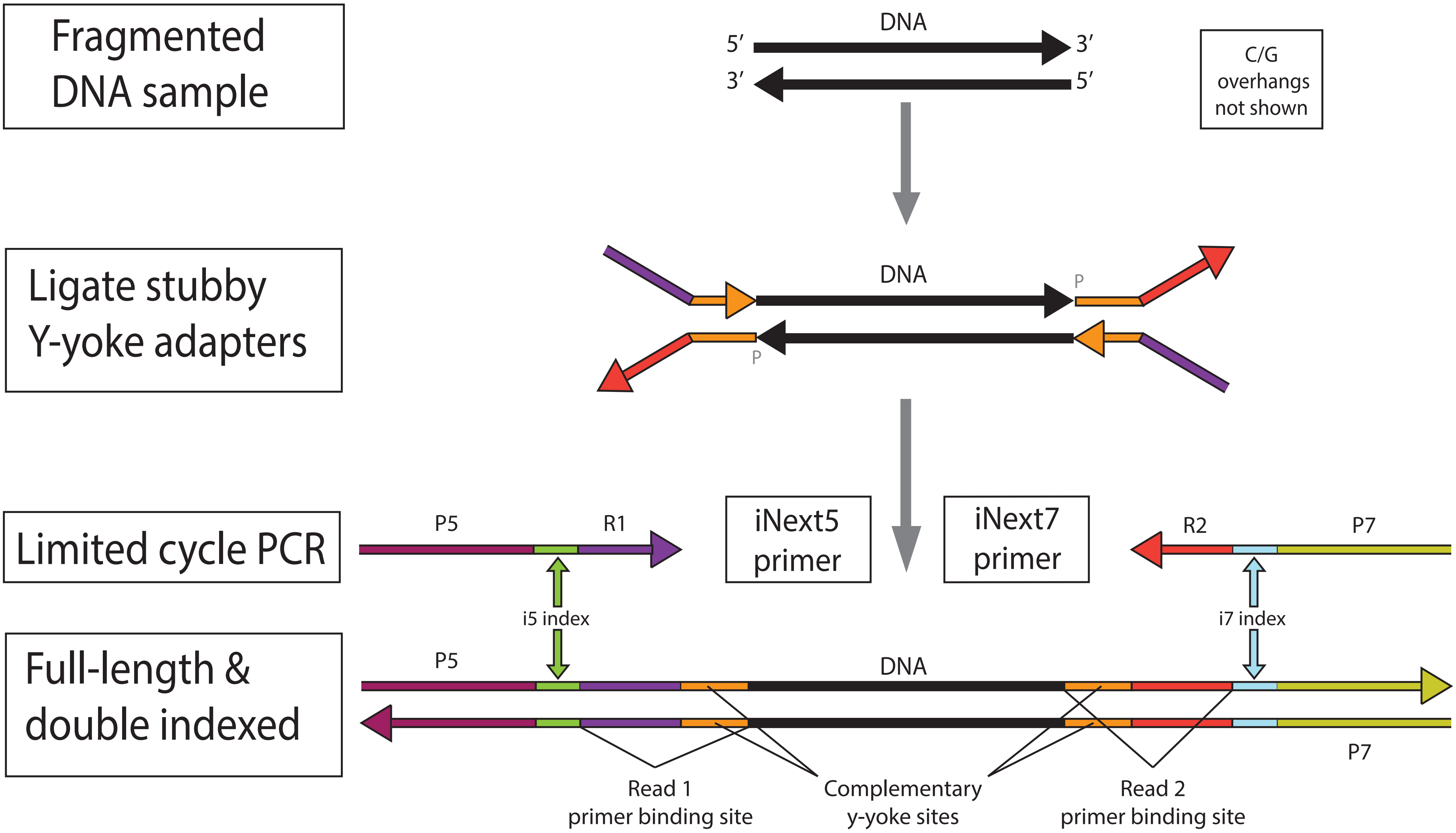

Supplement: Figure S2 — Color schemes and naming conventions follow those of Fig. 1 & S1. During library construction, sheared genomic DNA with C overhangs is ligated to stubby Y-yoke adapters with G overhangs (see Fig. 2). The C overhangs prevent chimeric ligation of genomic DNA molecules, and the G overhangs prevent ligation among adapters (adapter dimers). Adapters are phosphorylated (indicated with a “P” at the 5’ position), which allows ligation of stubs to genomic DNA. During limited cycle PCR, iNext5 and iNext7 primers anneal to the ends of the Y-yoke adapters to produce full-length, double-indexed molecules (cf. Fig. 2, S3, and S4). [file peerj-07-7755-s002.pdf]
