# Supplementary figures and images for: Adapterama I: universal stubs and primers for 384 unique dual-indexed or 147,456 combinatorially-indexed Illumina libraries (iTru & iNext)
Source: PeerJ. 2019 Oct 11;7:e7755. doi: 10.7717/peerj.7755 (PMC6791352; doi:10.7717/peerj.7755)

# Addition of Double-Index with PCR

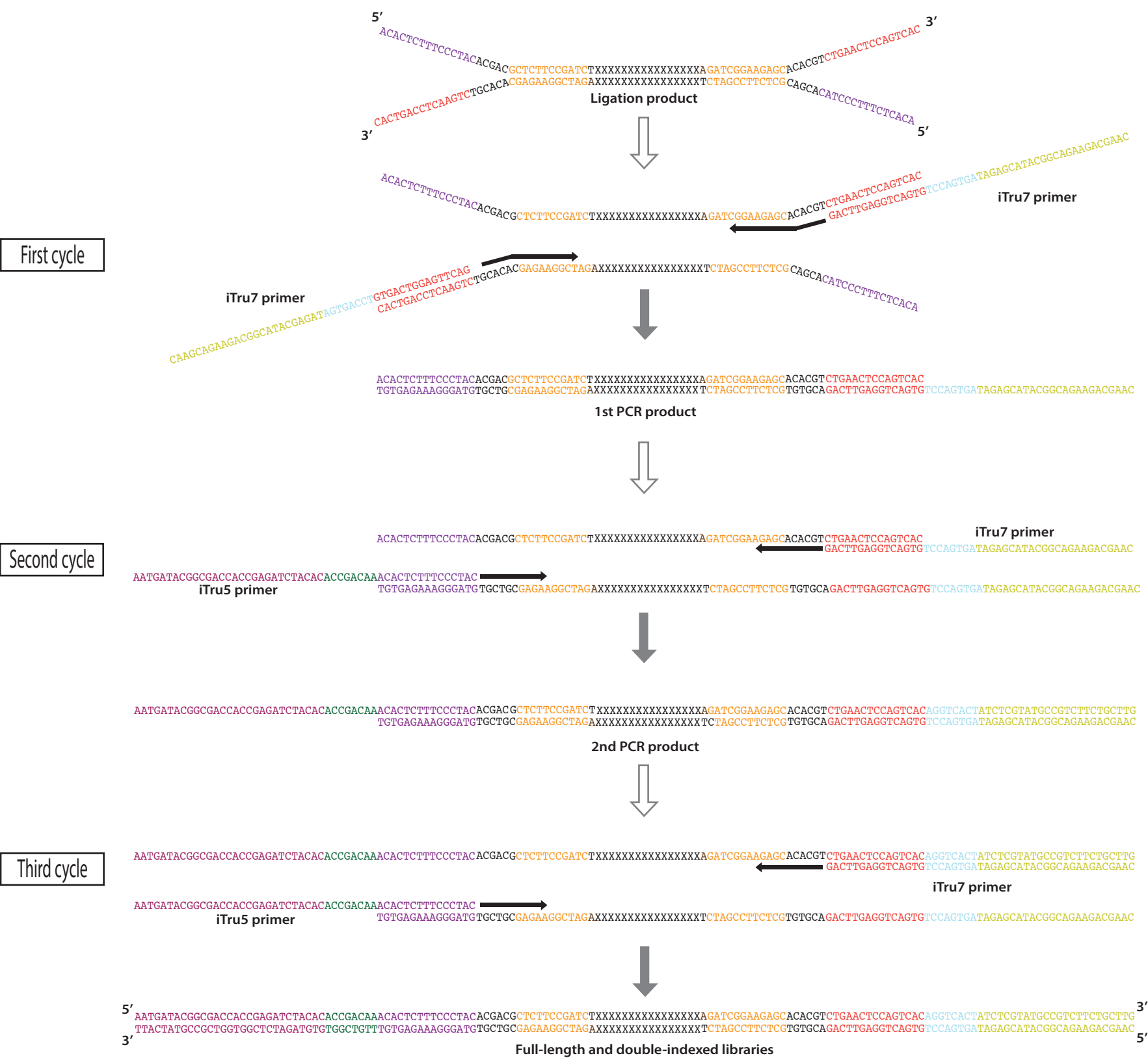

Supplement: Figure S3 — During PCR, adapter-ligated DNA molecules (i.e., the ligation products) react with index containing primers to create the double-indexed, full-length library molecules. During the first PCR cycle, only the iTru7 primer binds to the denatured strand at the 3′end of the template molecules. The iTru5 primer has the same sequence and orientation as the 5′ends of the molecule, and therefore cannot anneal during the first cycle. The product of the first cycle, an abbreviated single-indexed molecule, creates a sequence complementary to the iTru5 primer. During the second cycle, both primers are able to anneal to a denatured strand, creating a dual-indexed, truncated molecule with an overhang at the 3′end on the bottom strand. In the third cycle, a full-length, dual-indexed (i5 index on top strand, i7 on bottom) library-prepared molecule is made. [file peerj-07-7755-s003.pdf]

# Complete Library Molecule TruSeq (iTru) vs. Nextera (iNext)

iTru

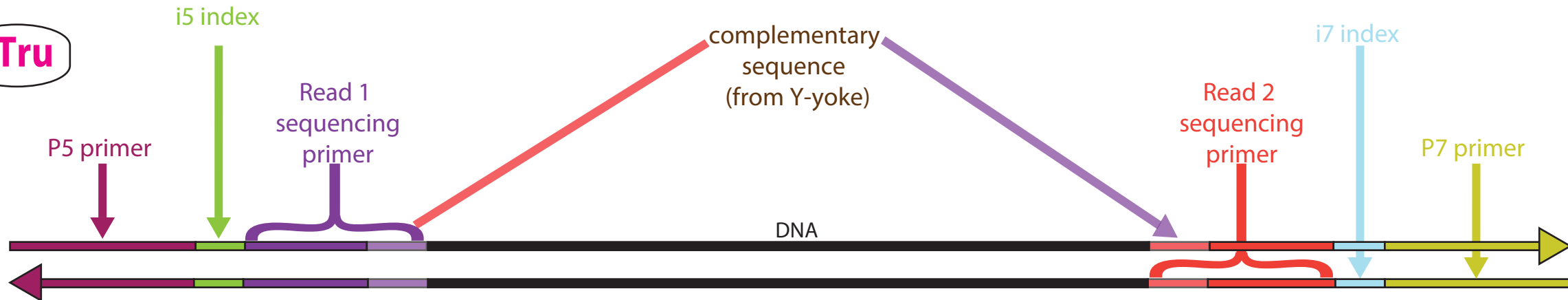

iNext

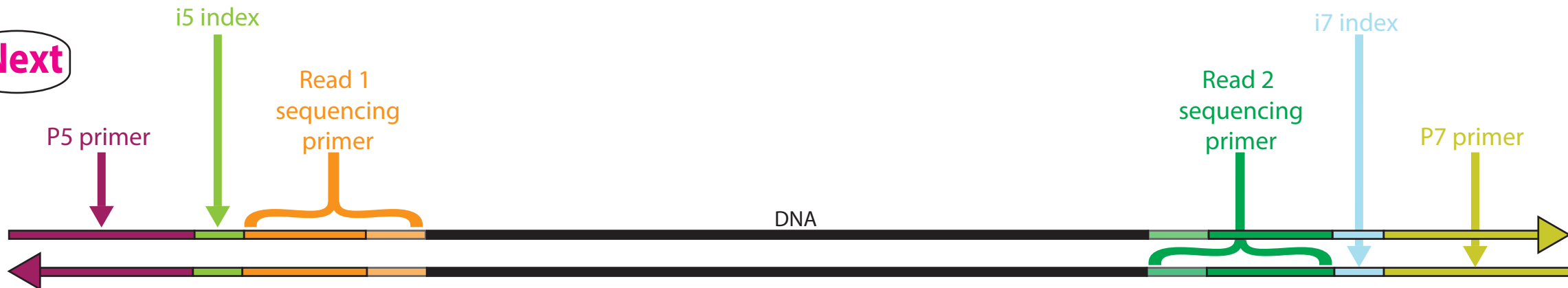

Supplement: Figure S4 — Complete double-stranded library molecules are illustrated. The color schemes used previously have been simplified so that the Y-yoke portions of Read1 (R1) and Read2 (R2) are now simply shown as lighter colors of the non-complementary regions. Although functionally equivalent, the R1 and R2 regions of iTru and iNext have no sequence similarity. In contrast, the P5 and P7 regions are identical. Although the iTru and iNext indexing regions (i5 and i7) are illustrated in the same colors and draw from the same pool of tags (Faircloth & Glenn, 2012), there is no correspondence in numbering (i.e., iTru5_01_A index & iNext5_01_A index). [file peerj-07-7755-s004.pdf]

# Complete Library Molecule and Sequencing Reads

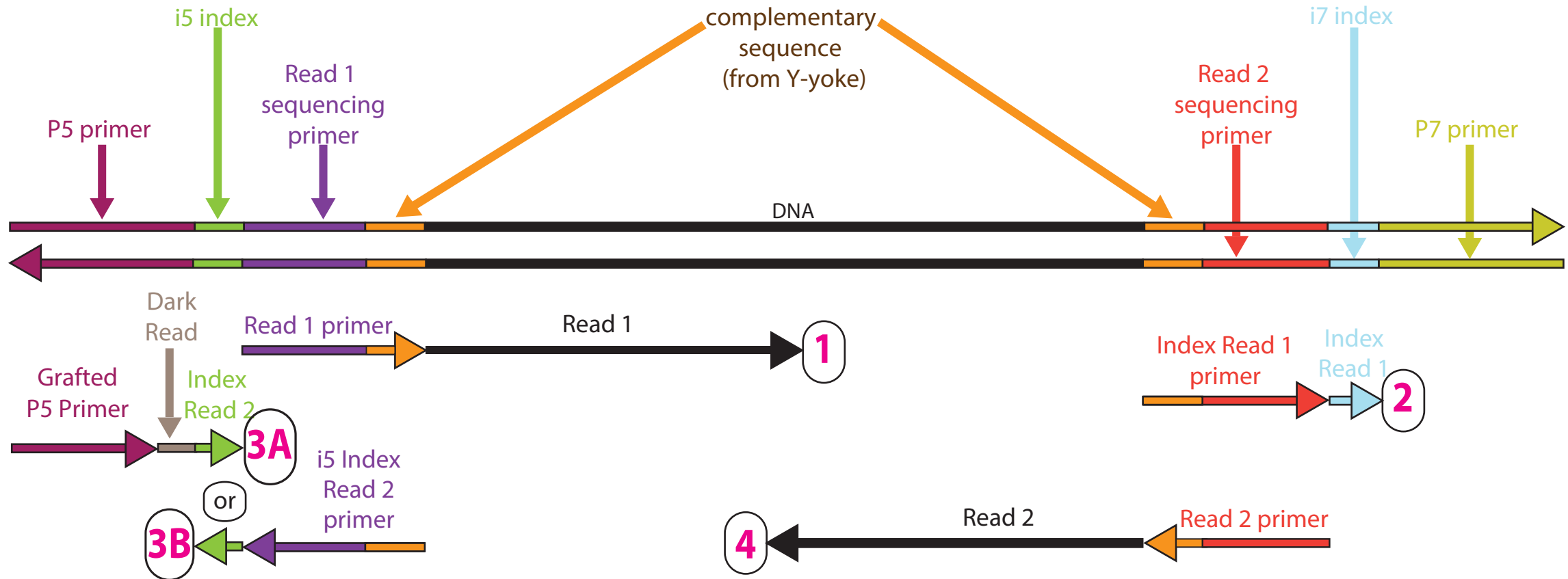

Supplement: Figure S6 — The color scheme here is similar to those used in Figs. 1 and 3, S1, S2, and S3. Reads work similarly for iNext. [file peerj-07-7755-s006.pdf]

# Sequencing Reads from Libraries Lacking an i5

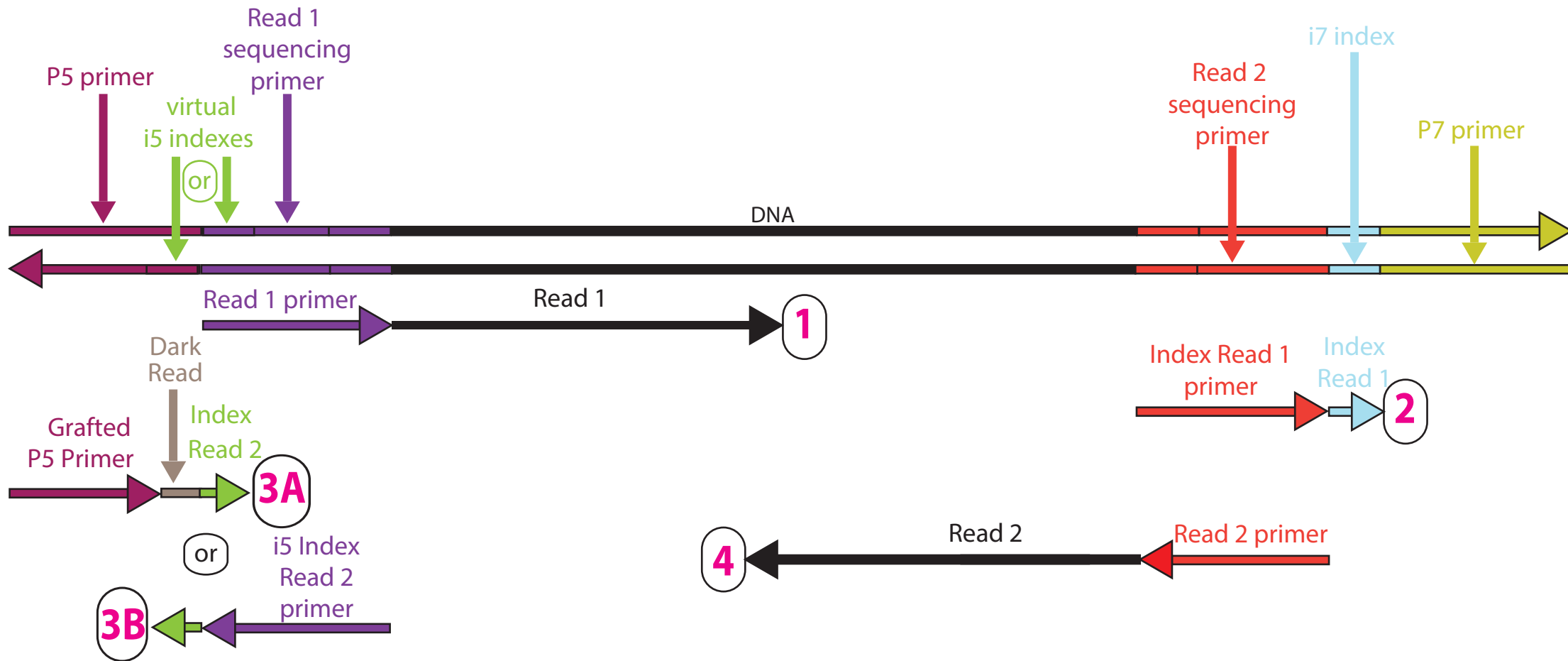

Supplement: Figure S7 — Illumina still supports libraries with a single index; the i7 index (i.e., Indexing Read1) is always used in these instances. If libraries of this type are mixed with iTru, or any other dual-indexing libraries, and both index sequencing reads are obtained from the pool, an i5 sequence will be generated, but different strands and thus positions will be sequenced based on which instrument (indexing read2 primer) is used. The i5 sequence obtained will be GTGTAGAT from NextSeq and MiniSeq, whereas the sequence ACACTCTT is obtained from MiSeq and HiSeq ≤2,500 instruments. HiSeq ≥3,000 instruments initially generate the sequence GTGTAGAT, but that is reverse complemented to ATCTACAC by Illumina software. Because all Nextera-type libraries are dual indexed, there is no similar situation for Nextera or iNext libraries. [file peerj-07-7755-s007.pdf]

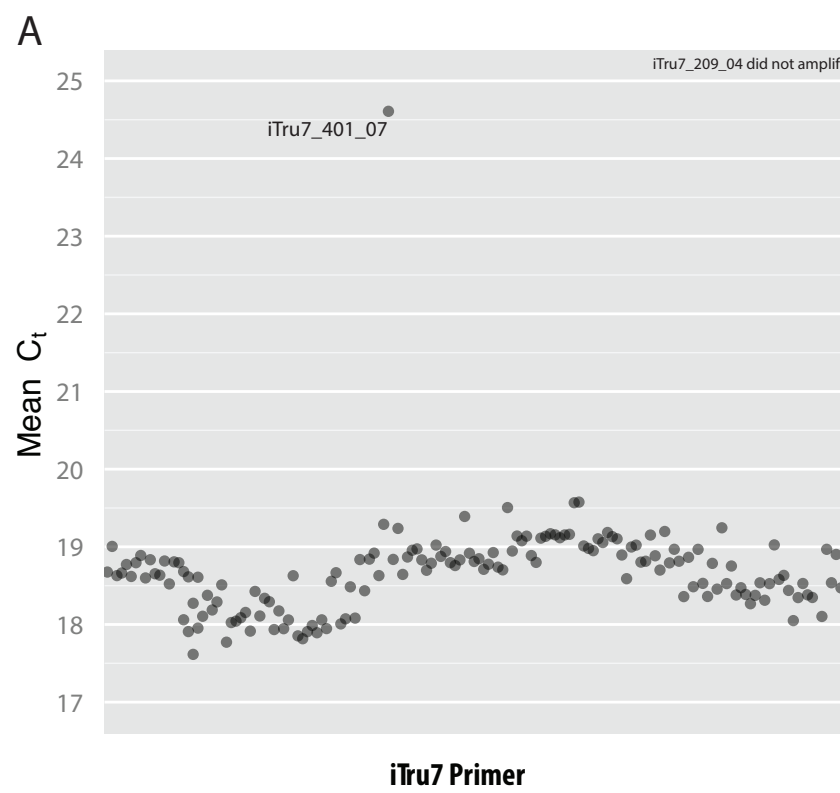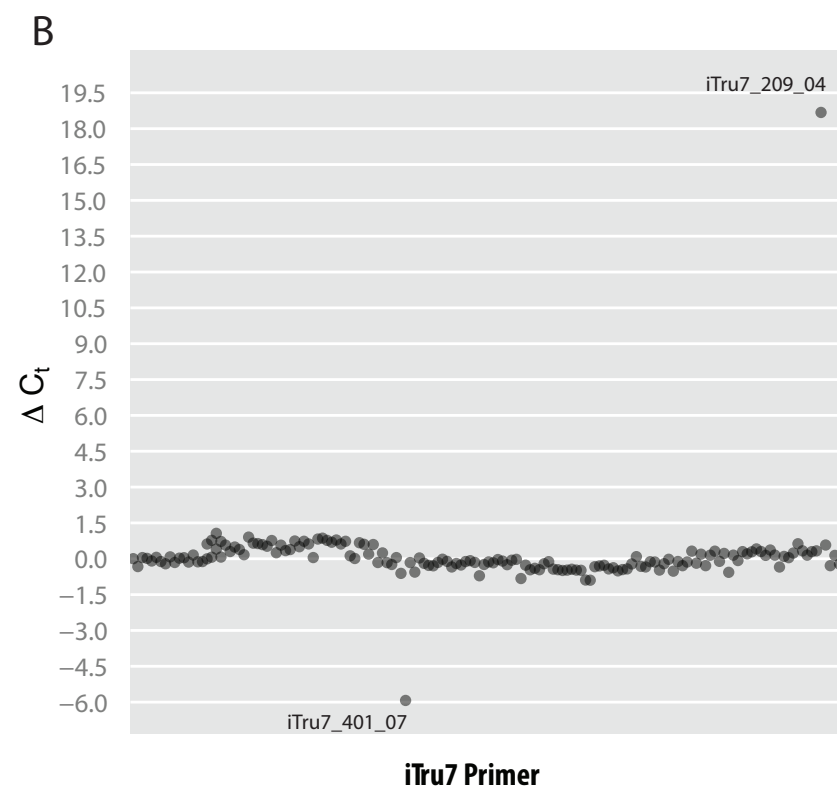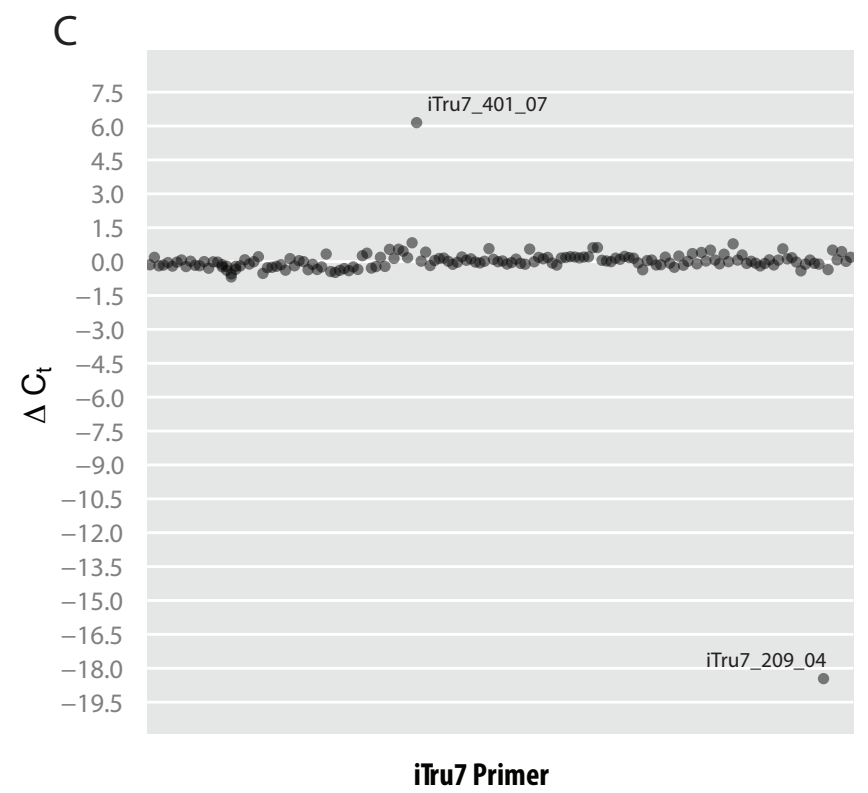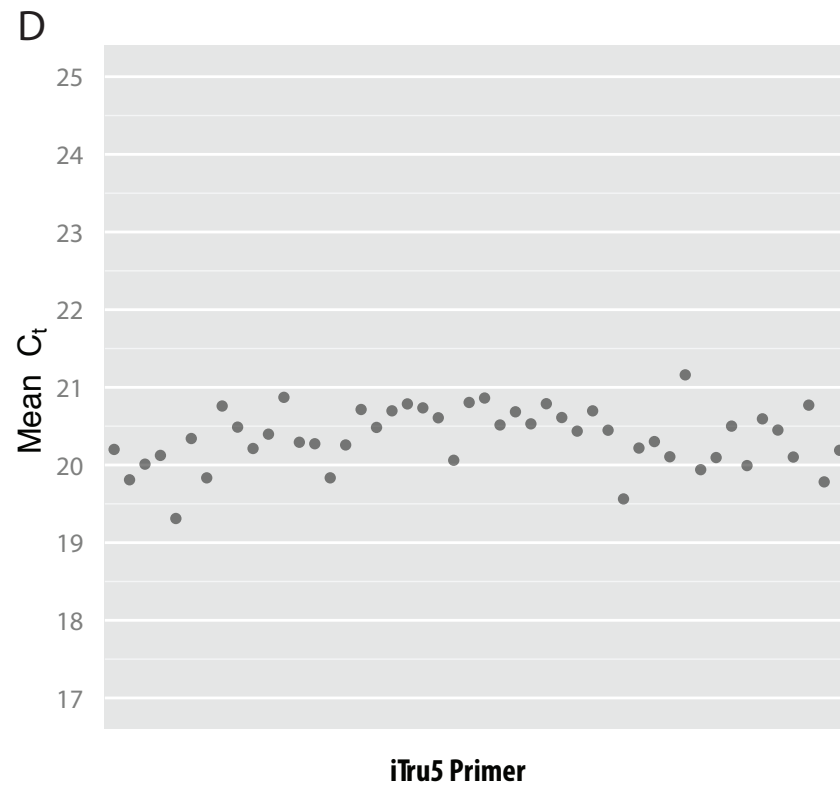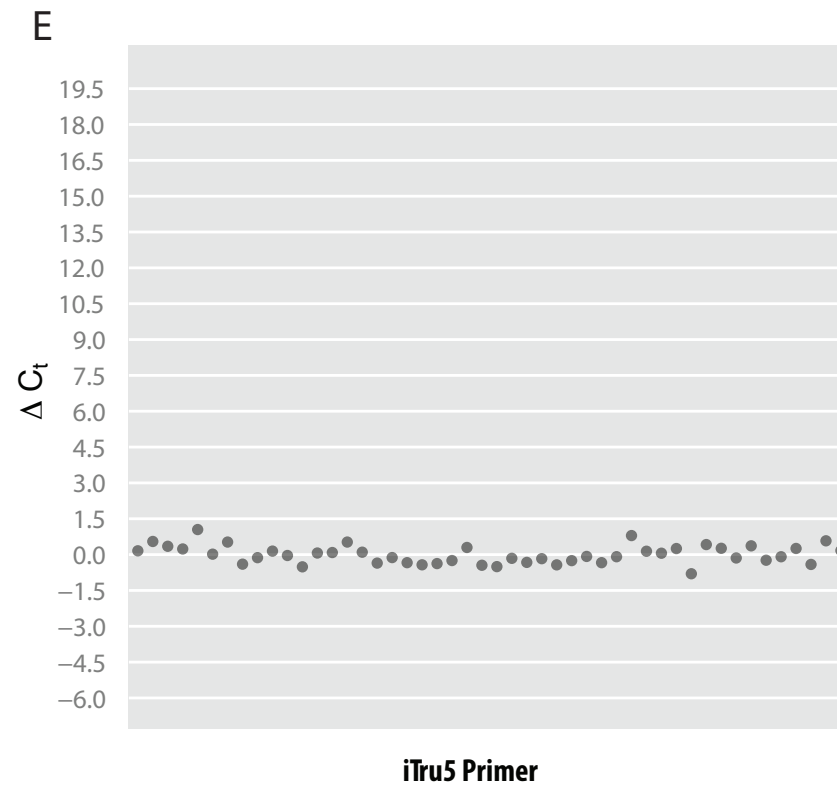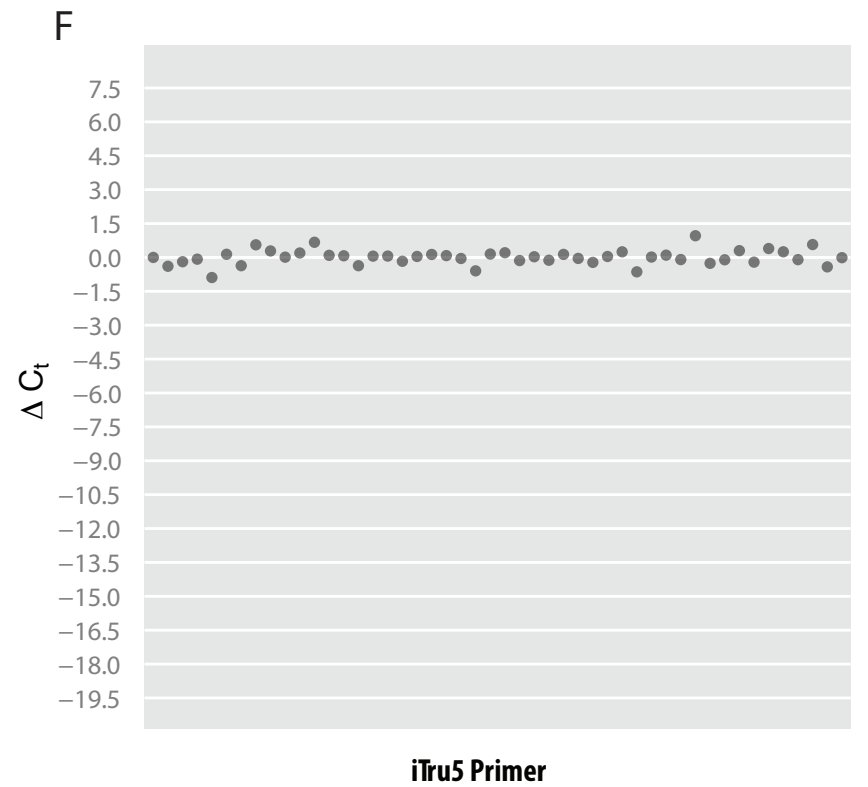

Supplement: Figure S8 — The iTru7_401_07 primer performed poorly during the first qPCR test, and we retested this same primer from a new aliquot of oligos. The iTru7_401_07 primer performed normally during this second retest. [file peerj-07-7755-s008.pdf]

Reads

1,200,000

1,000,000

800,000

600,000

iTru\_01

iTru\_02

iTru\_03

iTru\_04

iTru\_05

iTru\_06

iTru\_07

iTru\_08

iTru\_09

iTru\_10

iTru\_11

iTru\_12

Library

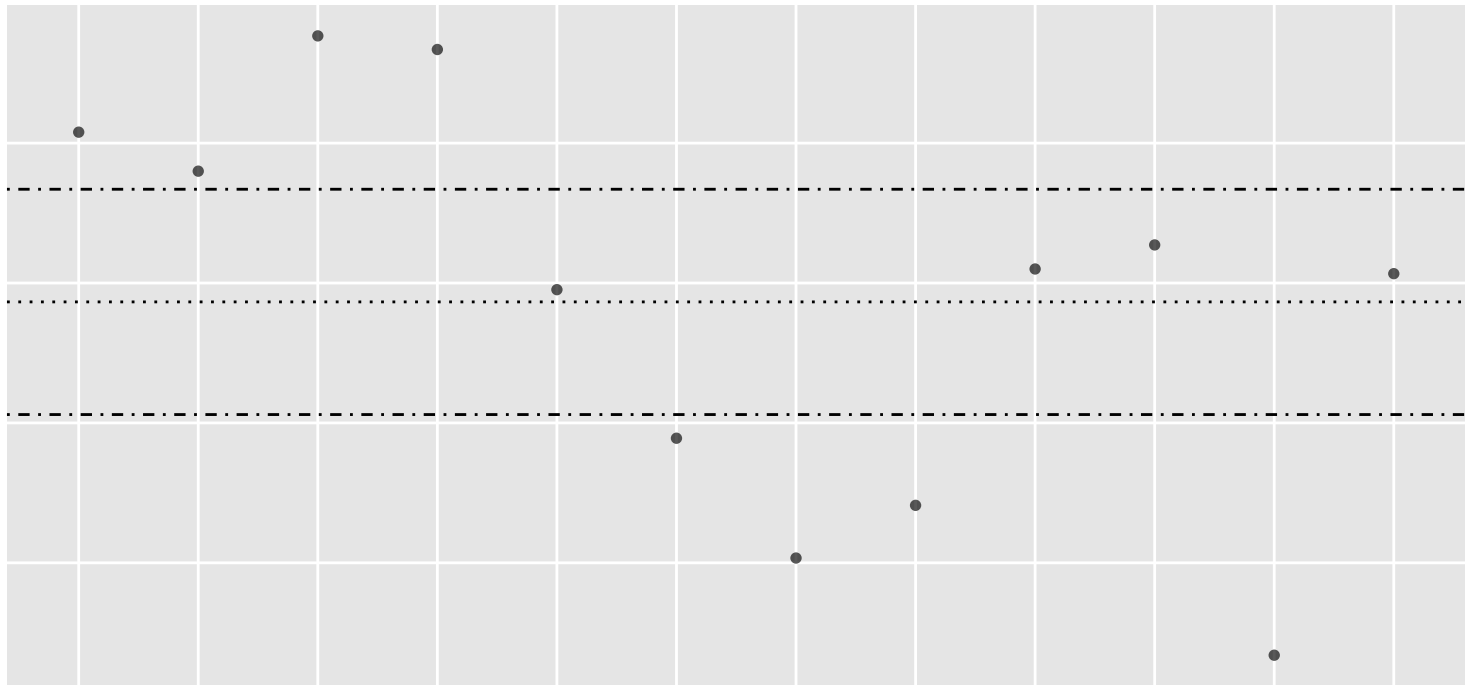

Supplement: Figure S9 [file peerj-07-7755-s009.pdf]

A.

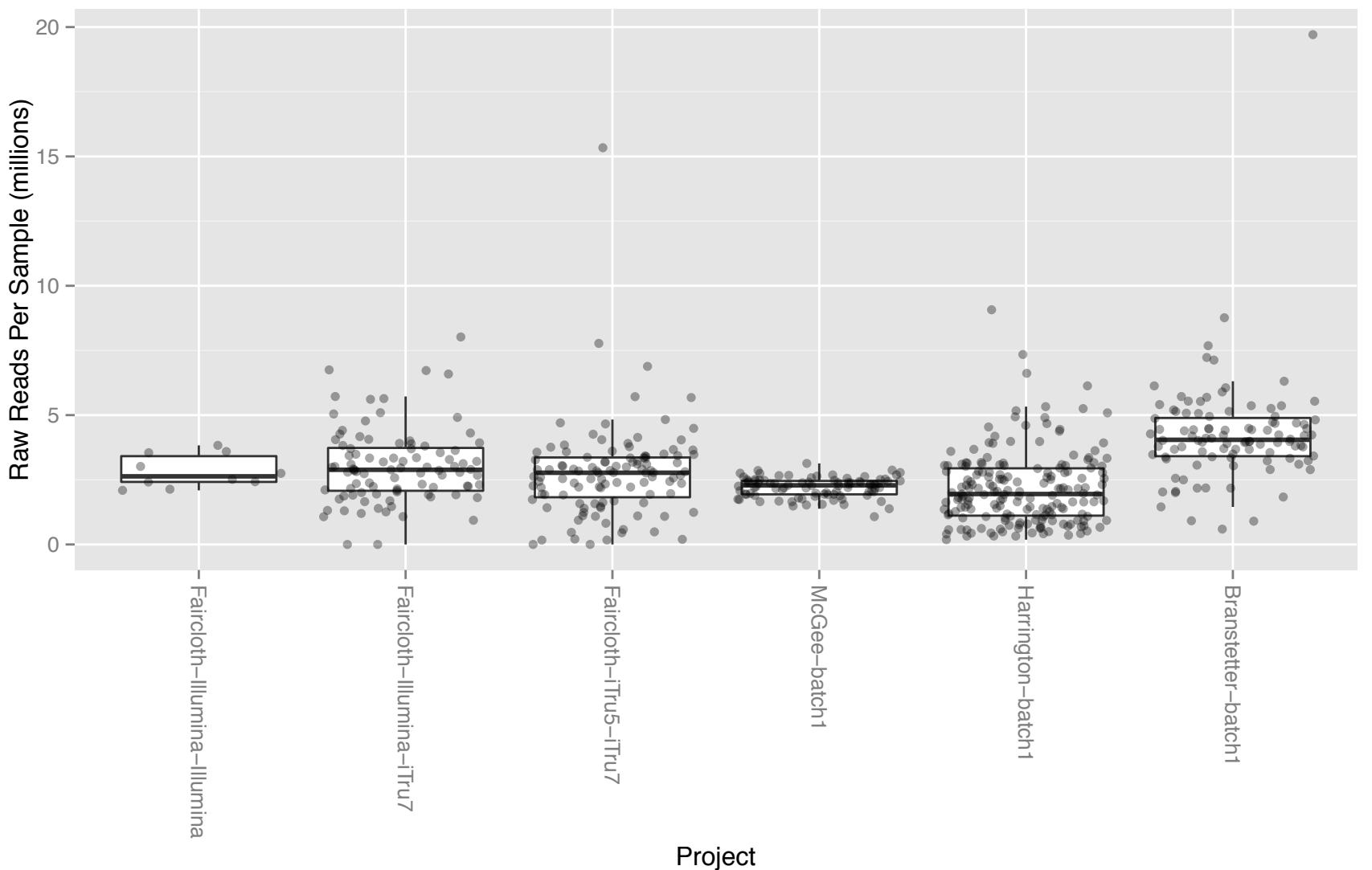

B.

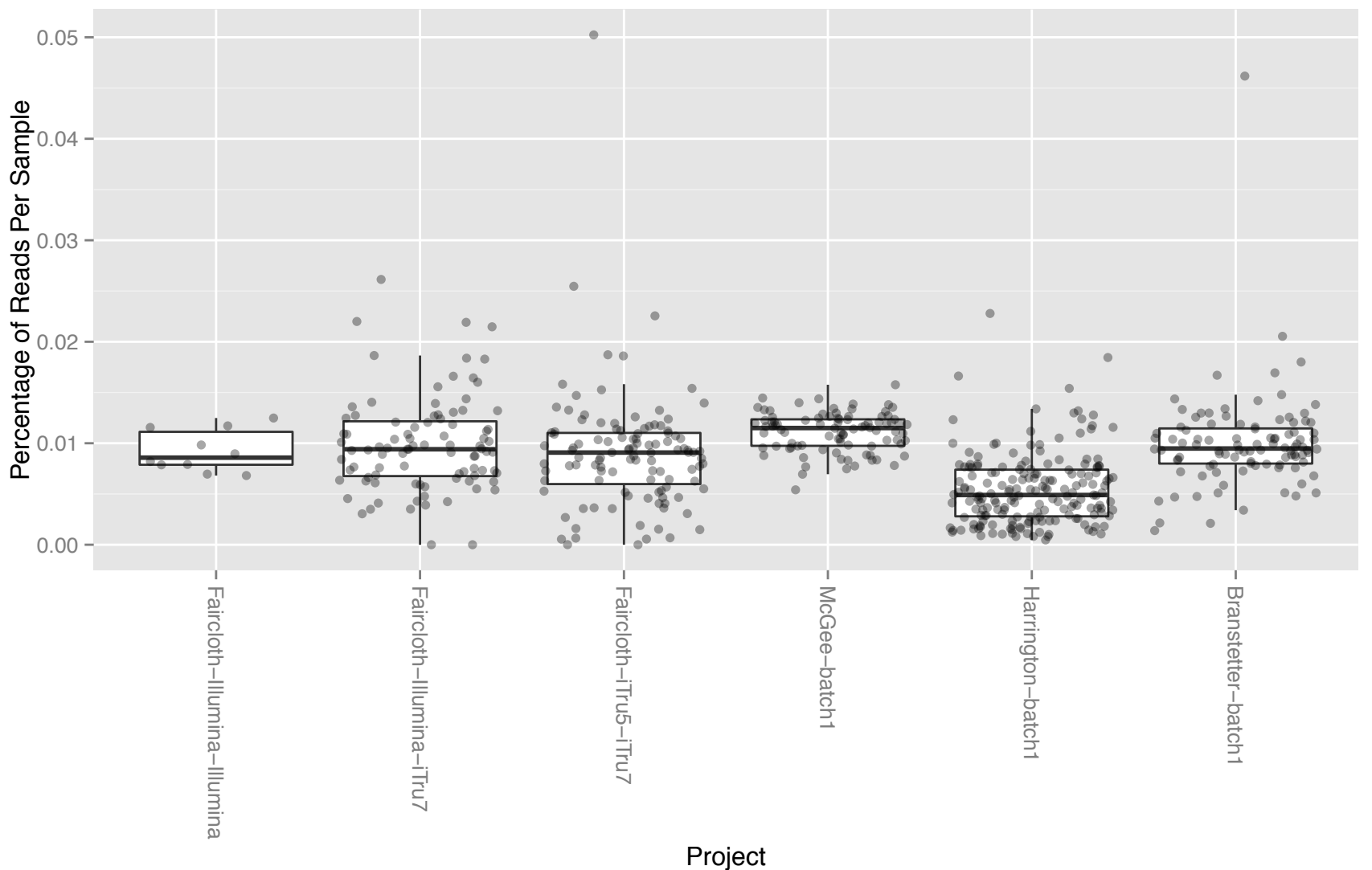

Supplement: Figure S10 [file peerj-07-7755-s010.pdf]

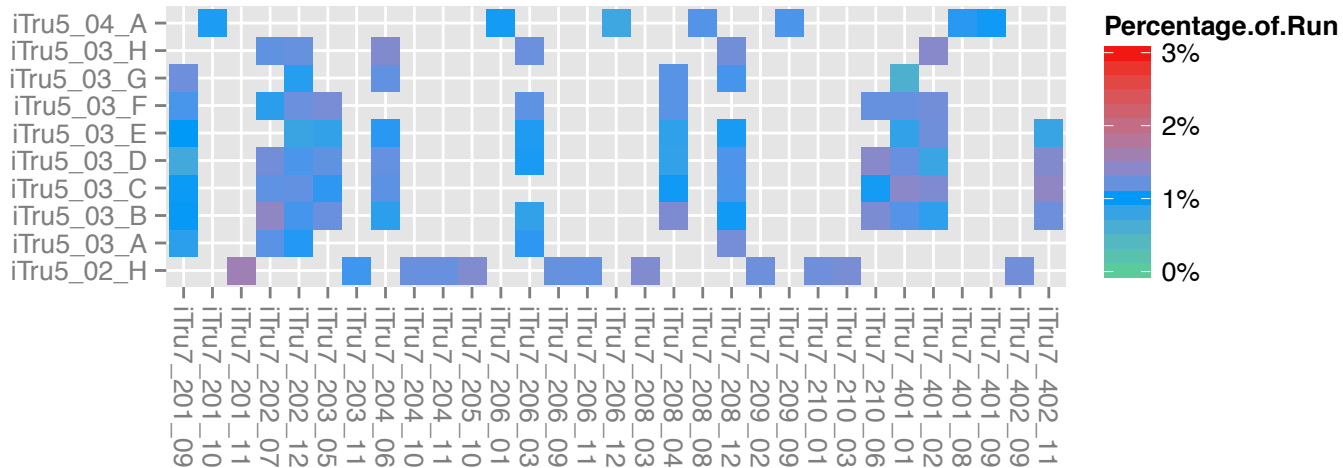

Supplement: Figure S11 — Data were generated from a partial, PE150, Illumina NextSeq High Output run, and the target for each sample was 1.0% of the total reads generated across the partial run (blue). The heat map shows deviations from the optimal percentage. [file peerj-07-7755-s011.pdf]

A.

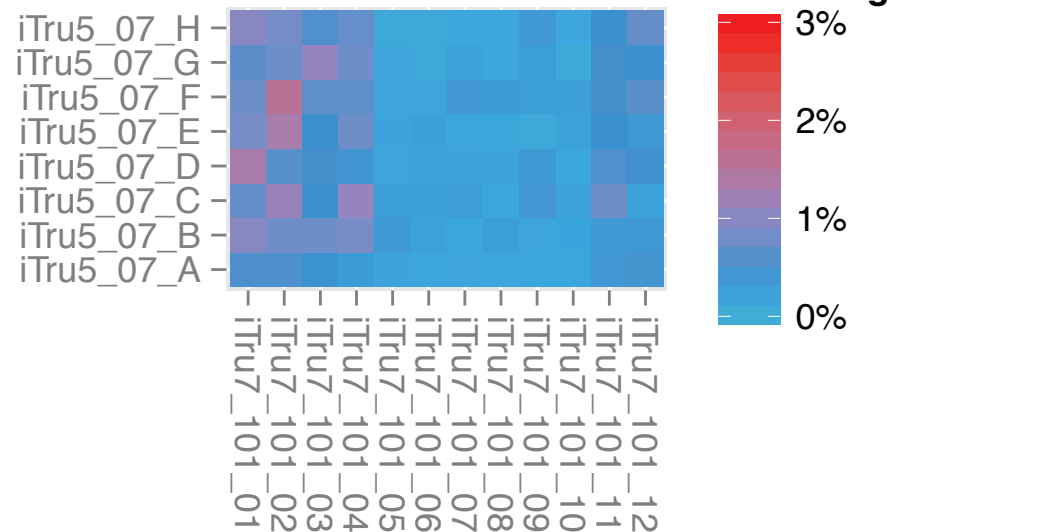

B.

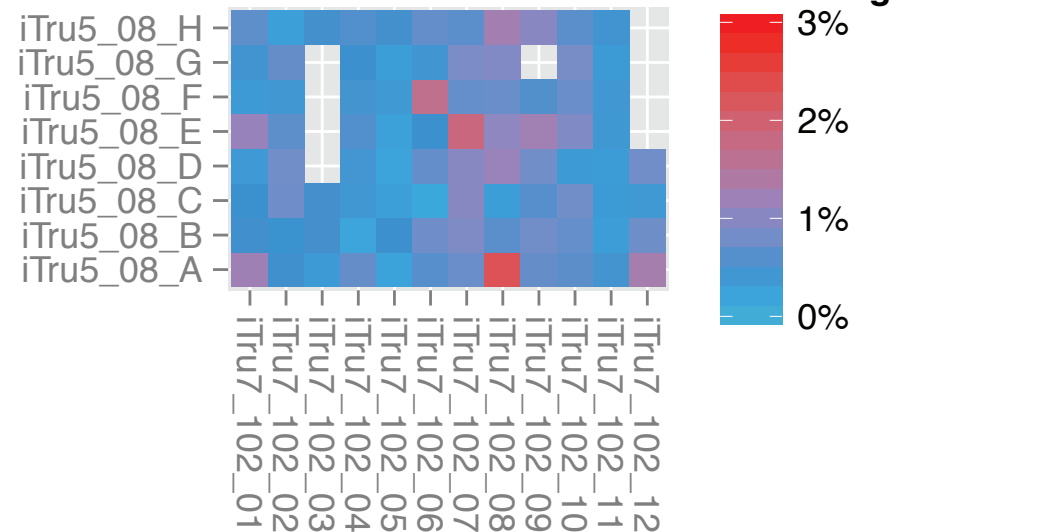

Supplement: Figure S12 — Data were generated from a partial, PE150, Illumina NextSeq High Output run, and the target for each sample was 0.5% of the total reads generated across the partial run (blue). The heat map shows deviations from the optimal percentage. [file peerj-07-7755-s012.pdf]

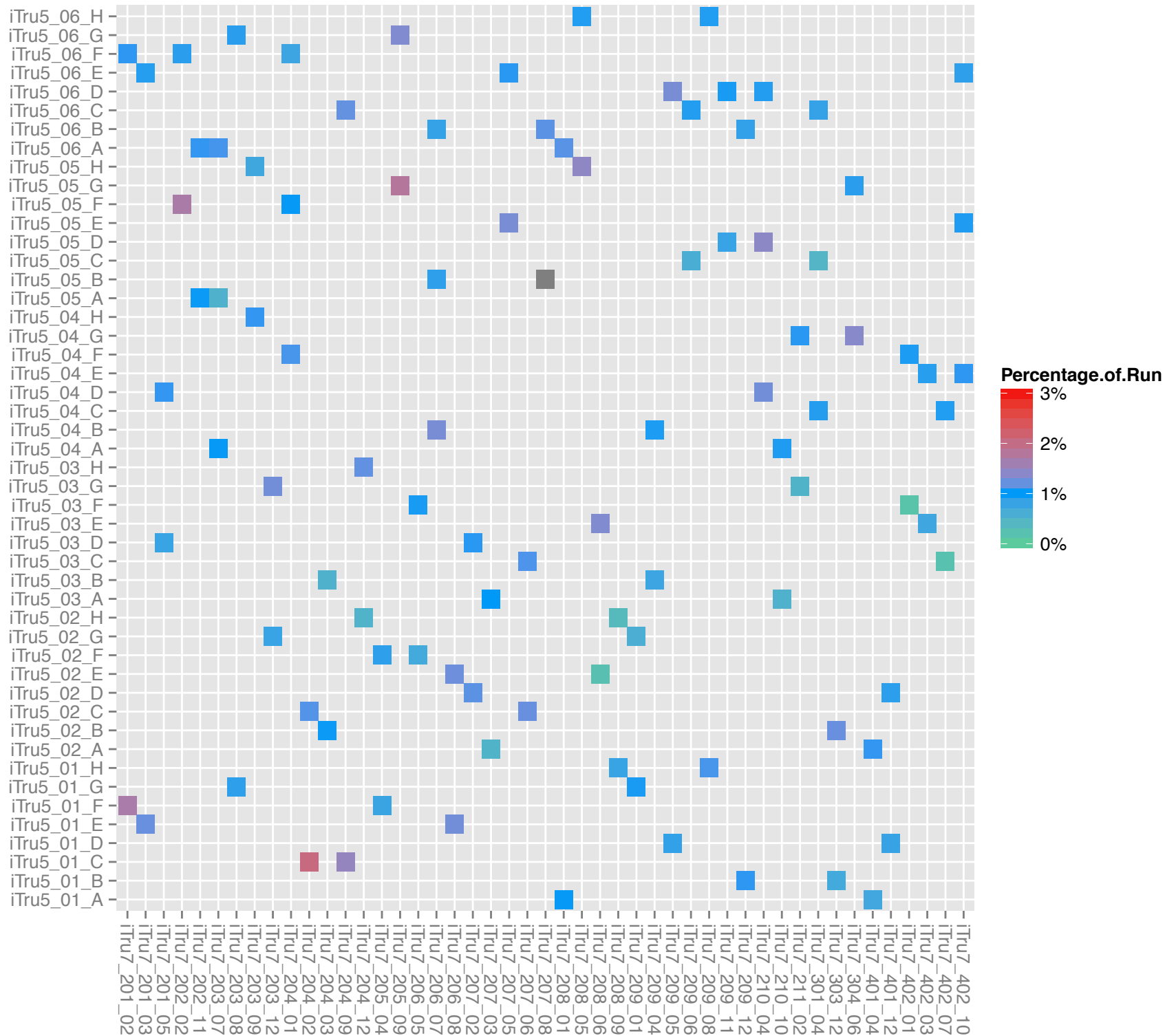

Supplement: Figure S13 — Data were generated from one lane of PE125 sequencing on an Illumina 2,500, and the target for each sample was 1% of the total reads generated across the entire run (blue). The heat map shows deviations from the optimal percentage. [file peerj-07-7755-s013.pdf]

A.

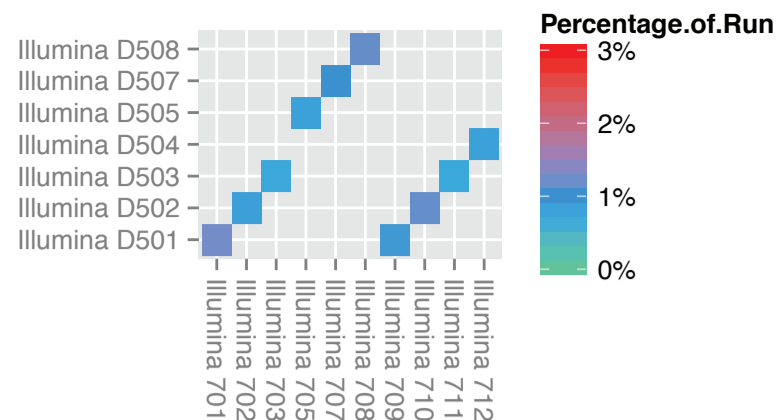

B.

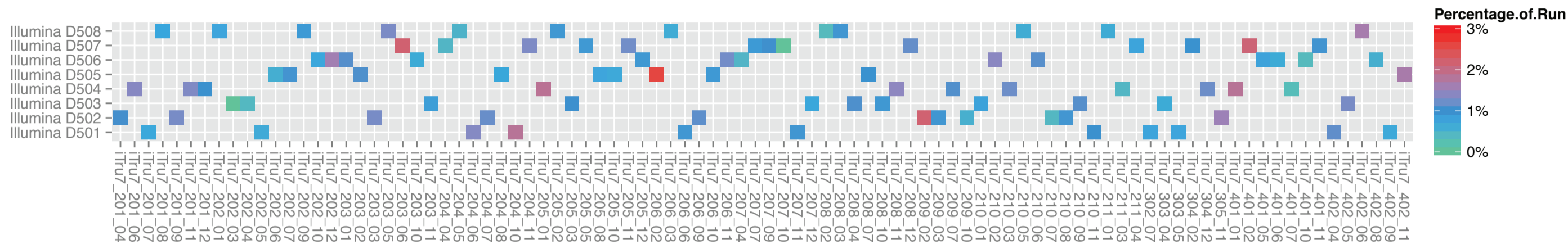

C.

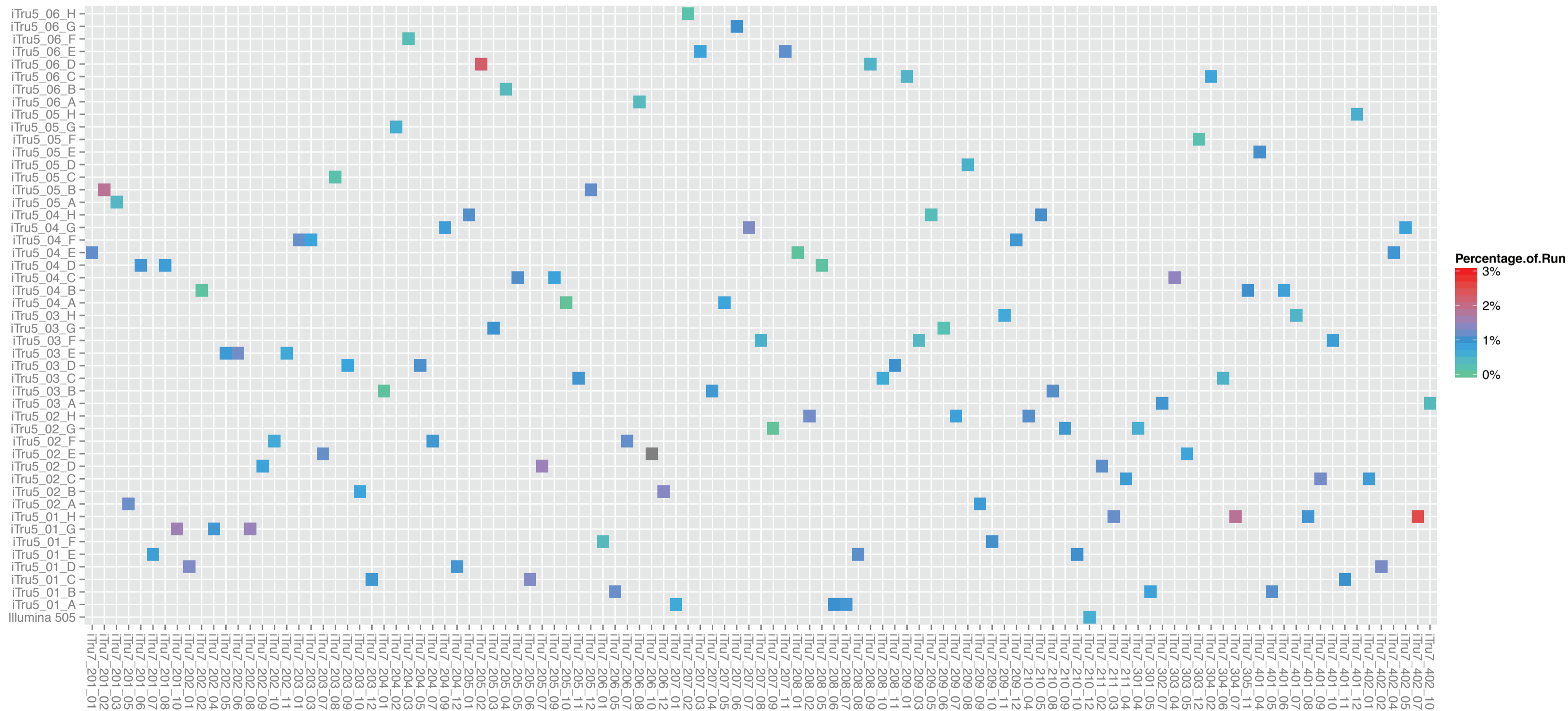

Supplement: Figure S14 — Data were generated from two lanes of PE150 sequencing on an Illumina 1500 in Rapid Run mode, and the target for each sample was 1% of the total reads generated across the entire run (blue). The heat map shows deviations from the optimal percentage. [file peerj-07-7755-s014.pdf]

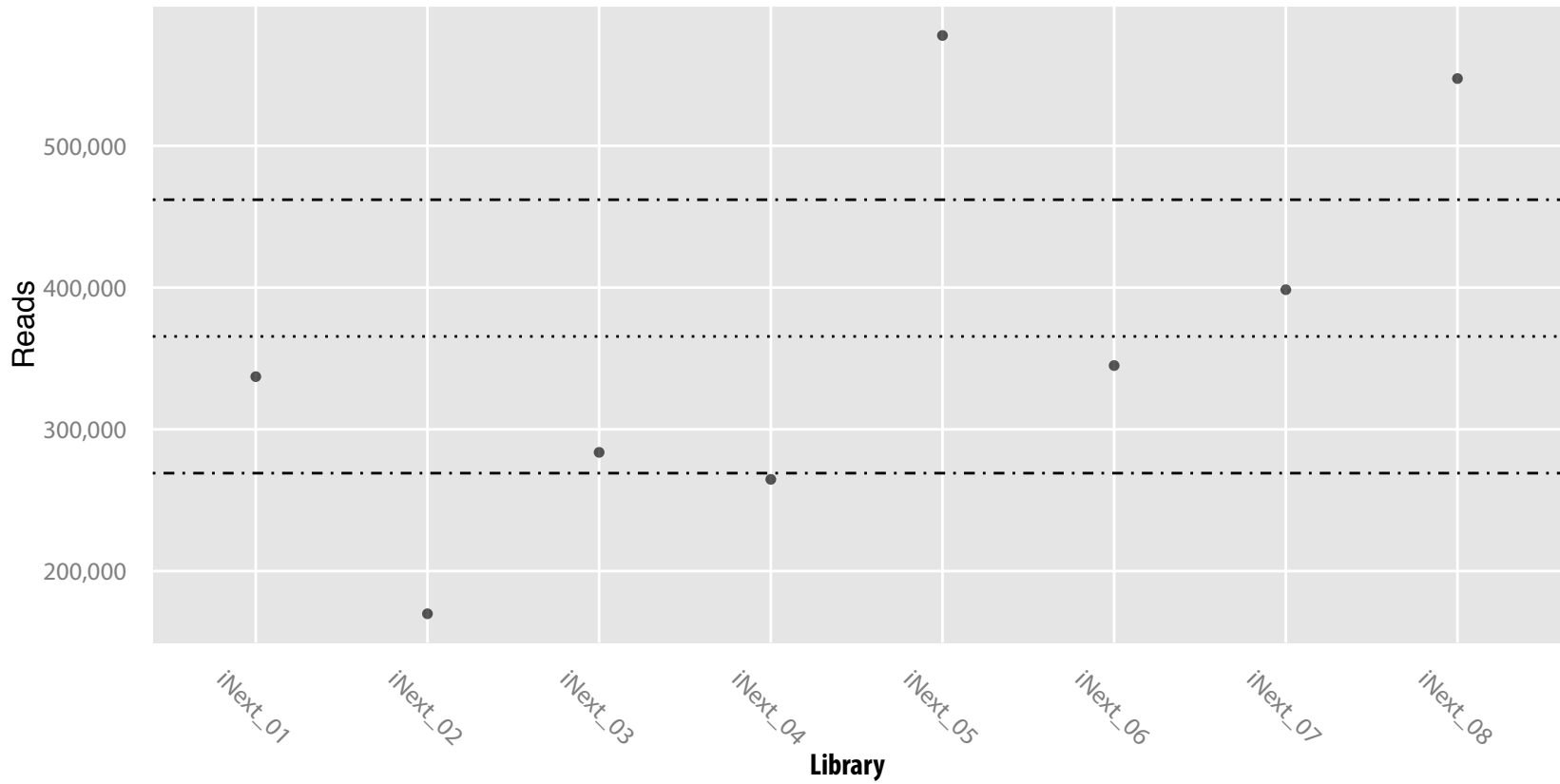

Supplement: Figure S15 [file peerj-07-7755-s015.pdf]
