# Supplementary material for: Adapterama I: universal stubs and primers for 384 unique dual-indexed or 147,456 combinatorially-indexed Illumina libraries (iTru & iNext)
Source: PeerJ. 2019 Oct 11;7:e7755. doi: 10.7717/peerj.7755 (PMC6791352; doi:10.7717/peerj.7755)
Supplement: Figure S5 — Figure shows the sequence tags used by Illumina on the P7 side of the library construct in the Illumina TruSeq HT kits (D###) and the Illumina TruSeq (TS-##) kits. Note that several of the edit distances within and between each set of sequence tags are ≤3 (e.g. D702 vs. D710; D705 vs. TS-15), which precludes the use of edit-distance error correction algorithms to recover sequence tags containing a sequencing error. [file peerj-07-7755-s005.pdf]

	D701	D702	D703	D704	D705	D706	D707	D708	D709	D710	D711	D712	TS-1	TS-2	TS-3	TS-4	TS-5	TS-6	TS-7	TS-8	TS-9	TS-10	TS-11	TS-12	TS-13	TS-14	TS-15	TS-16	TS-18	TS-19	TS-20	TS-21	TS-22	TS-23	TS-25	TS-27
D701	–	–	–	–	–	–	–	–	–	–	–	–	–	–	–	–	–	–	–	–	–	–	–	–	–	–	–	–	–	–	–	–	–	–	–	–
D702	7	–	–	–	–	–	–	–	–	–	–	–	–	–	–	–	–	–	–	–	–	–	–	–	–	–	–	–	–	–	–	–	–	–	–	–
D703	7	6	–	–	–	–	–	–	–	–	–	–	–	–	–	–	–	–	–	–	–	–	–	–	–	–	–	–	–	–	–	–	–	–	–	–
D704	5	8	6	–	–	–	–	–	–	–	–	–	–	–	–	–	–	–	–	–	–	–	–	–	–	–	–	–	–	–	–	–	–	–	–	–
D705	5	4	6	7	–	–	–	–	–	–	–	–	–	–	–	–	–	–	–	–	–	–	–	–	–	–	–	–	–	–	–	–	–	–	–	–
D706	5	7	6	3	5	–	–	–	–	–	–	–	–	–	–	–	–	–	–	–	–	–	–	–	–	–	–	–	–	–	–	–	–	–	–	–
D707	5	5	6	5	5	5	–	–	–	–	–	–	–	–	–	–	–	–	–	–	–	–	–	–	–	–	–	–	–	–	–	–	–	–	–	–
D708	5	5	7	4	6	3	5	–	–	–	–	–	–	–	–	–	–	–	–	–	–	–	–	–	–	–	–	–	–	–	–	–	–	–	–	–
D709	6	6	3	6	7	6	6	7	–	–	–	–	–	–	–	–	–	–	–	–	–	–	–	–	–	–	–	–	–	–	–	–	–	–	–	–
D710	6	2	6	8	4	7	6	5	5	–	–	–	–	–	–	–	–	–	–	–	–	–	–	–	–	–	–	–	–	–	–	–	–	–	–	–
D711	5	4	6	7	6	6	5	3	7	3	–	–	–	–	–	–	–	–	–	–	–	–	–	–	–	–	–	–	–	–	–	–	–	–	–	–
D712	5	5	5	5	5	6	6	7	4	5	7	–	–	–	–	–	–	–	–	–	–	–	–	–	–	–	–	–	–	–	–	–	–	–	–	–
TS-1	4	5	5	7	3	5	4	6	6	4	5	5	–	–	–	–	–	–	–	–	–	–	–	–	–	–	–	–	–	–	–	–	–	–	–	–
TS-2	6	6	4	5	7	5	4	5	4	6	7	5	5	–	–	–	–	–	–	–	–	–	–	–	–	–	–	–	–	–	–	–	–	–	–	–
TS-3	5	5	6	7	5	5	4	4	5	5	5	6	4	4	–	–	–	–	–	–	–	–	–	–	–	–	–	–	–	–	–	–	–	–	–	–
TS-4	6	6	4	6	6	6	5	6	5	5	6	5	4	4	5	–	–	–	–	–	–	–	–	–	–	–	–	–	–	–	–	–	–	–	–	–
TS-5	6	5	5	6	5	5	5	6	5	4	6	5	3	4	4	5	–	–	–	–	–	–	–	–	–	–	–	–	–	–	–	–	–	–	–	–
TS-6	6	5	4	5	6	5	5	7	5	5	7	4	4	4	5	5	6	–	–	–	–	–	–	–	–	–	–	–	–	–	–	–	–	–	–	–
TS-7	6	6	3	4	6	4	4	5	4	6	7	5	5	3	6	5	3	6	–	–	–	–	–	–	–	–	–	–	–	–	–	–	–	–	–	–
TS-8	5	5	5	7	4	5	4	6	6	5	5	5	4	4	4	5	3	5	5	–	–	–	–	–	–	–	–	–	–	–	–	–	–	–	–	–
TS-9	6	6	4	6	3	4	5	6	6	6	6	5	2	4	4	5	6	3	4	6	–	–	–	–	–	–	–	–	–	–	–	–	–	–	–	–
TS-10	6	6	4	4	6	5	6	6	4	5	6	4	5	4	6	4	4	6	3	4	6	–	–	–	–	–	–	–	–	–	–	–	–	–	–	–
TS-11	6	6	3	5	6	4	6	6	4	6	6	4	4	4	4	4	3	4	6	2	3	5	–	–	–	–	–	–	–	–	–	–	–	–	–	–
TS-12	6	6	5	7	5	6	4	7	5	6	6	6	5	3	5	5	3	5	5	5	4	4	4	–	–	–	–	–	–	–	–	–	–	–	–	–
TS-13	5	6	5	6	3	7	5	6	5	6	6	4	4	6	5	3	4	4	4	5	5	4	4	5	–	–	–	–	–	–	–	–	–	–	–	–
TS-14	4	7	4	4	4	3	6	5	6	6	5	6	4	5	4	4	4	5	3	4	4	4	4	5	5	–	–	–	–	–	–	–	–	–	–	–
TS-15	5	5	5	7	2	5	5	5	6	6	5	5	4	5	5	4	6	3	4	5	4	4	6	4	5	4	–	–	–	–	–	–	–	–	–	–
TS-16	5	6	5	5	7	6	5	6	5	5	4	6	6	6	4	5	4	5	4	2	5	4	5	5	5	5	5	–	–	–	–	–	–	–	–	–
TS-18	6	4	6	6	5	6	6	5	6	3	3	6	4	6	6	5	6	4	5	5	5	6	4	6	5	5	5	5	–	–	–	–	–	–	–	–
TS-19	4	7	7	4	6	5	3	5	5	7	6	4	6	6	6	5	6	7	4	4	4	5	4	6	5	3	5	6	4	–	–	–	–	–	–	–
TS-20	6	7	5	6	7	5	5	7	5	6	6	7	5	6	5	5	5	6	5	5	4	6	6	5	3	4	5	7	5	6	–	–	–	–	–	–
TS-21	5	5	6	6	4	4	6	6	6	5	4	7	5	6	7	6	6	7	5	6	7	7	6	6	5	4	5	6	6	6	5	–	–	–	–	–
TS-22	5	5	4	7	5	5	5	6	5	5	6	5	5	3	4	5	6	5	6	6	4	6	4	6	4	5	5	5	6	5	5	4	–	–	–	–
TS-23	7	6	6	5	5	4	5	5	6	6	7	5	5	3	6	5	7	5	6	6	5	7	5	5	4	6	5	5	5	7	6	4	6	–	–	–
TS-25	5	5	4	6	4	6	4	6	4	5	6	3	4	4	4	4	6	6	5	6	6	4	5	5	6	4	5	5	5	5	4	7	6	4	–	–
TS-27	3	7	5	6	4	5	6	6	6	6	6	6	4	6	5	6	7	7	7	5	4	6	6	6	6	4	3	6	4	6	5	5	5	6	4	–
