# Supplementary material for: Adapterama I: universal stubs and primers for 384 unique dual-indexed or 147,456 combinatorially-indexed Illumina libraries (iTru & iNext)
Source: PeerJ. 2019 Oct 11;7:e7755. doi: 10.7717/peerj.7755 (PMC6791352; doi:10.7717/peerj.7755)
Supplement: File S15 [file peerj-07-7755-s030.zip › Count_both_indexes/Easiest_Read_Counter_Directions.doc]

Directions for obtaining the index reads from a file of Illumina fastq.gz reads on a Mac:

If you have a fastq.gz file of undetermined reads from an Illumina sequencer (i.e., the indexes did not match the sample sheet), you can use this to determine what indexes are most common among those reads. It won’t always work, because the sequence headers can be modified by the people running your Illumina sequencer, but this will work most of the time. It was made for dual indexes, but seems to work correctly for single-indexes as well. The Core Code was written by Brant Faircloth (@LSU) and was slightly updated with extra comments & print commands by Travis Glenn ([travisg@uga.edu](mailto:travisg@uga.edu)).

Written: 14 October 2014	Latest Update: 14 August, 2015

1) Go to your Documents folder & make a new folder named “CountTheIndexes” [note: without the quotes]
2) Place a copy of the gzipped file of reads (e.g., YourUndeterminedReadFile.fastq.gz) into your new folder, along with these 3 files:
   a. countIndexes.py
   b. testCountIndexes.py
   c. test2CountIndexes.py
   d. UndeterminedTestFile.fastq.gz
3) Open Terminal [it is a program that lives in Applications -> Utilities]
4) Navigate your way to the folder where the files from above exist:

$ cd Documents

$ cd CountTheIndexes

5) Ensure you see the 4 files in there:

$ ls

you should see the 4 files listed by name

6) Confirm that everything is working by typing the following command:

$ python testCountIndexes.py

If it doesn’t work, ensure you typed everything exactly. Output should look like this:

```
You are now searching the file UndeterminedTestFile.fastq.gz

Here is the sum of all sequences in the file with dual barcodes:
1000

Here are the 50 most common barcode combinations in the file:

 [('AGCGATAG+TCAGAGCC', 28),
 ('GGGGGGGG+AGATCTCG', 23),
 ('CTGAAGCT+ACGTCCTG', 17),
 ('AAGGCGTT+AGGATAGG', 16),
… [45 more]
 ('ATTACTCG+AGGCTATA', 6)]
```

7) Optional: Confirm that your reads have indexes incorporated into the headers

$ gunzip -c YourUndeterminedReadFile.fastq.gz | head -n 40

output will look like this (sequence & qual scores truncated; index shown in red)

@NS500216:19:H0R72AGXX:1:11101:19715:1045 1:N:0:GTCTAGGT+CTGGAGTA

TTGCTNTATTTATTTATTCATTG...

+

<AAAA#FFFFFAFFFAFFFFFFF...

8) Optional: Copy the output from #7 above & paste it into a text file saved as testCountMyIndexes.fastq.gz (saved into the folder CountTheIndexes); and run:

$ python test2CountIndexes.py

You should see output similar to #6 above, but with your actual indexes, from the first 10 sequences. To double-check that all is well, you can compare the indexes the program finds & outputs, vs. the 10 input reads. This is just a small sample of reads, but it quickly confirms that the program will work on your input file and return correct output.

9) Change the name of your large file with undetermined reads:

From: YourUndeterminedReadFile.fastq.gz

To: countMyIndexes.fastq.gz

10) Type the following command:

$ python CountIndexes.py

11) You should see output similar to #6 above, but with the specific indexes for your input file. Note, if your file is large, it can take quite a while for this to run. On my 2008 Mac Pro (2.8 GHz Intel Xeon – only one processor is used, so it doesn’t matter how many you have), it takes about 7 minutes per GB of filesize (so a 5 GB file is ~35 minutes to finish). If you open up Activity Monitor, you should see that one of your cores is ~100% busy with Python 2.7 while it is running.
12) After you have determined what indexes are most frequent within the file, then you can either modify your sample sheet & demultiplex again using bcl2fastq (best) or pull out reads with specific indexes (e.g., using the sep_index.py program)
13) Note: This program works with dual indexes or single indexes. If you have dual index files and you want to count only the i7 or i5 indexes, then use the files in the folders: i7_only_index_counter or the i5_only_index_counter. They work exactly as these do, I have just extended the names so that they describe which index is being counted. I have also made a test file with only 2 sequences in it so that you can very easily see what is going on & confirm that all is well.
